# Unraveling Risk Genes of COVID-19 by Multi-Omics Integrative Analyses

**DOI:** 10.3389/fmed.2021.738687

**Published:** 2021-09-07

**Authors:** Ancha Baranova, Hongbao Cao, Fuquan Zhang

**Affiliations:** ^1^School of Systems Biology, George Mason University, Manassas, VA, United States; ^2^Research Centre for Medical Genetics, Moscow, Russia; ^3^Institute of Neuropsychiatry, The Affiliated Brain Hospital of Nanjing Medical University, Nanjing, China; ^4^Department of Psychiatry, The Affiliated Brain Hospital of Nanjing Medical University, Nanjing, China

**Keywords:** GWAS, COVID-19, TWAS, eQTL, mQTL

## Abstract

**Objectives:** Uncovering the genetic basis of COVID-19 may shed insight into its pathogenesis and help to improve treatment measures. We aimed to investigate the host genetic variants associated with COVID-19.

**Methods:** The summary result of a COVID-19 GWAS (9,373 hospitalized COVID-19 cases and 1,197,256 controls) was obtained from the COVID-19 Host Genetic Initiative GWAS meta-analyses. We tested colocalization of the GWAS signals of COVID-19 with expression and methylation quantitative traits loci (eQTL and mQTL, respectively) using the summary data-based Mendelian randomization (SMR) analysis. Four eQTL and two mQTL datasets were utilized in the SMR analysis, including CAGE blood eQTL data (*n* = 2,765), GTEx v7 blood (*n* = 338) and lung (*n* = 278) eQTL data, Geuvadis lymphoblastoid cells eQTL data, LBC-BSGS blood mQTL data (*n* = 1,980), and Hannon blood mQTL summary data (*n* = 1,175). We conducted a transcriptome-wide association study (TWAS) on COVID-19 with precomputed prediction models of GTEx v8 eQTL in lung and blood using S-PrediXcan.

**Results:** Our SMR analyses identified seven protein-coding genes (*TYK2, IFNAR2, OAS1, OAS3, XCR1, CCR5*, and *MAPT*) associated with COVID-19, including two novel risk genes, *CCR5* and tau-encoding *MAPT*. The TWAS revealed four genes for COVID-19 (*CXCR6, CCR5, CCR9*, and *PIGN*), including two novel risk genes, *CCR5* and *PIGN*.

**Conclusion:** Our study highlighted the functional relevance of some known genome-wide risk genes of COVID-19 and revealed novel genes contributing to differential outcomes of COVID-19 disease.

## Introduction

Severe acute respiratory syndrome coronavirus 2 (SARS-CoV-2) and resultant COVID-19 have created a public health crisis worldwide. The majority of infected persons are either affected mildly or stay asymptomatic. It was reported that ~10–20% of people with COVID-19 infection need hospitalization (1). Hypertension, obesity, and diabetes are among the common comorbidities of hospitalized patients (2). Patients with older age or medical complications tend to have severe symptoms. However, some young and seemingly healthy individuals may also have serious outcomes from the virus infection. As the symptoms, severity, and prognosis of the disease are highly variable, host genetics may influence human's susceptibility to COVID-19, in a similar manner as it was noted for other infectious diseases (3).

The need in elucidating the genetic drivers of the development of COVID-19 is urgent as it may allow novel insights into its pathogenesis. Host Genetic Initiative (HGI) is one of the global efforts to promote human genetic variance research of COVID-19 by platform building, analytical activities, and data sharing (4). Genome-wide association studies (GWASs) have been conducted worldwide to characterize gene variants defining the susceptibility and severity of the COVID-19. In particular, Severe Covid-19 GWAS Group has identified two loci associated with SARS-CoV-2 related respiratory failure, including the chr3p21.31 locus with multiple genes encoding chemokine receptors and the chr9q34.2 locus with the blood type gene ABO (5). Pairo-Castineira et al. also revealed a set of genetic variants enriched in COVID-19 patients admitted to intensive care units [6]. This set highlighted chr3p21.31, chr12q24.13 (*OAS1, OAS2*, and *OAS3*), chr19p13.2 (*TYK2*), chr19p13.3 (*DPP9*), and chr21q22.1 (*IFNAR2*) (6). These two studies provide valuable evidence for the genetic basis of COVID-19. Both of the studies analyzed the datasets collected by HGI at the early stages of the project.

To get a clear understanding of these GWAS outputs and gain more insight into the SARS-CoV-2 pathophysiology, we performed summary data-based Mendelian randomization (SMR) and transcriptome-wide association analyses. We prioritized the genes co-localized with the COVID-19 GWAS hits and mapped additional involved genes. The resultant list of genes suggests potential therapeutic targets for symptomatic COVID-19.

## Methods

### The COVID-19 Dataset and the Participants

The summary result of a COVID-19 GWAS (9,373 hospitalized COVID-19 cases and 1,197,256 controls, excluding 23andMe) was obtained from the COVID-19 Host Genetic Initiative (HGI) GWAS meta-analyses round 5 (Release Date: January 18, 2021) (4). All the participants were of European origins. Ethical approval had been obtained in all original studies. A more detailed description of the datasets is provided in the Supplementary File.

### Annotation of the COVID-19 GWAS Dataset

FUMA was used to map SNPs to genes and identify genomic regions independent of linkage disequilibrium (LD) (7). All genes located closer than 10 kb of each variant were mapped. Independent significant SNPs (IndSigSNPs) were extracted, according to criteria of significance at the genome level (*P* ≤ 5.0 × 10^−8^) and of independence (*r*^2^ < 0.6). For each group of IndSigSNPs, lead SNPs were identified when they were in LD with each other at *r*^2^ < 0.1 within a 500 Kb window. The merging of lead SNPs into genomic risk loci was performed when they were located at a distance of <500 kb from each other. Clumping was carried out according to the European 1,000 Genomes Project phase 3 reference panel, with the entire MHC locus being merged into one region (chr6:25-35Mb).

### SMR Analyses

Colocalization of GWAS signal with expression and methylation quantitative traits loci (eQTL and mQTL, respectively) was performed in a framework of the SMR v1.03 (8). In this, the GWAS summary result and eQTL data were used at the gene level to associate its expression level with a trait of interest. We utilized four eQTL and two mQTL datasets, including CAGE blood eQTL data (*n* = 2,765) (9), GTEx v7 blood (*n* = 338) and lung (*n* = 278) eQTL data (10), Geuvadis lymphoblastoid cells eQTL data (*n* = 373) (11), LBC-BSGS blood mQTL data (*n* = 1,980) (12), and Hannon blood mQTL summary data (*n* = 1,175) (13). Bonferroni procedure was employed to adjust *P*-values for multiple testing. Pleiotropic effects were sorted from the LD artifacts using the test for non-significant heterogeneity (P_HEIDI_ > 0.01) which is embedded in the SMR analysis workflow.

### TWAS Analyses

Putatively causal genes were prioritized by a TWAS procedure, which was conducted for the lung and the whole blood cells. The gene-level association results were calculated from GWAS summary statistics using S-PrediXcan (14, 15). We used precomputed prediction models of GTEx v8 (16) eQTL and LD references from http://predictdb.org/. Bonferroni procedure was employed to adjust *P*-values for multiple testing.

## Results

### Genomic Loci Identification of the COVID-19 GWAS

A total of six genomic loci were identified in the COVID-19 dataset, respectively (Table 1; Supplementary Figure 1, Table 1). The 3p21.31 locus contains the largest amount of association signals and genes (Supplementary Figure 2). A total of 20 genome-wide genes were detected for the COVID-19 GWAS (Table 1). These genes included *LIMD1, SLC6A20, LZTFL1, CCR9, FYCO1, CXCR6, XCR1, CCR3, FLT1P1, CCR1, UQCRC2P1, CCR2, LRRC2, VSTM2A, ABO, OAS1, OAS3, OAS2, DPP9*, and *IFNAR2*. Two of these genes, *FLT1P1* and *UQCRC2P1*, are non-coding genes.

**Table 1:** Genomic loci of the COVID-19 GWAS.

**CHR**	**BP**	**Band**	**SNP**	**Alleles**	**OR [95 CI]**	**P**	**Genes**
3	45889921	3p21.31	rs35081325	A/T	1.63 [1.53–1.73]	3.68E-54	LIMD1;SLC6A20;LZTFL1;CCR9;FYCO1;CXCR6;XCR1;CCR3;FLT1P1;CCR1;UQCRC2P1;CCR2;LRRC2
7	54647894	7p11.2	rs622568	A/C	1.17 [1.11–1.23]	3.64E-09	VSTM2A
9	136149229	9q34.2	rs920065566	C/T	0.89 [0.86–0.93]	4.42E-09	ABO
12	113357442	12q24.13	rs2660	G/A	1.12 [1.08–1.17]	2.01E-09	OAS1;OAS3;OAS2
19	4719443	19p13.3	rs2109069	G/A	1.16 [1.12–1.21]	2.94E-14	DPP9
21	34615210	21q22.11	rs13050728	T/C	0.85 [0.81–0.88]	7.44E-17	IFNAR2

*CHR, chromosome; BP, base position*.

### SMR Analyses of COVID-19

**Figure 1:**
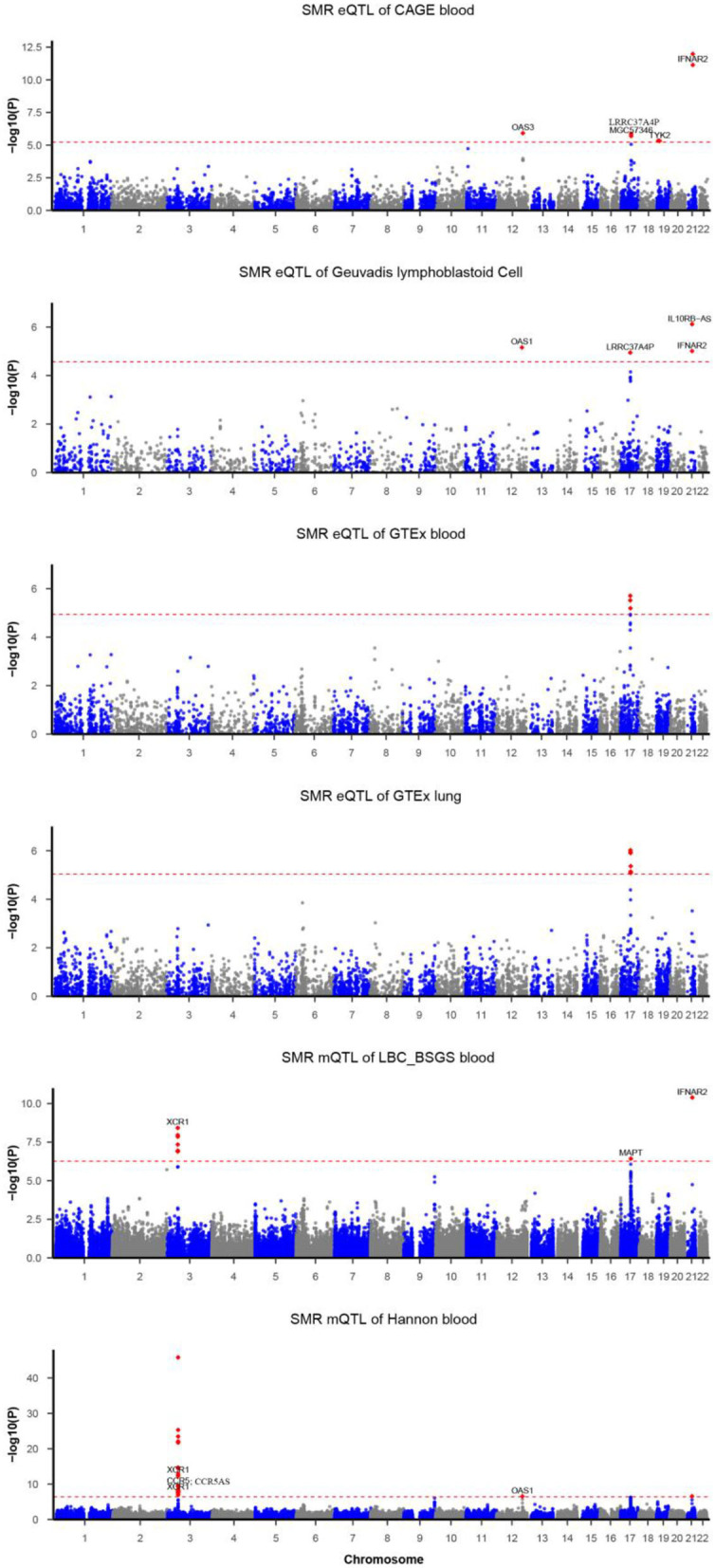
Summary data-based Mendelian randomization (SMR) analysis of COVID-19. Each horizontal dashed line denotes a genome-wide significance level adjusted by Bonferroni.

**Figure 2:**
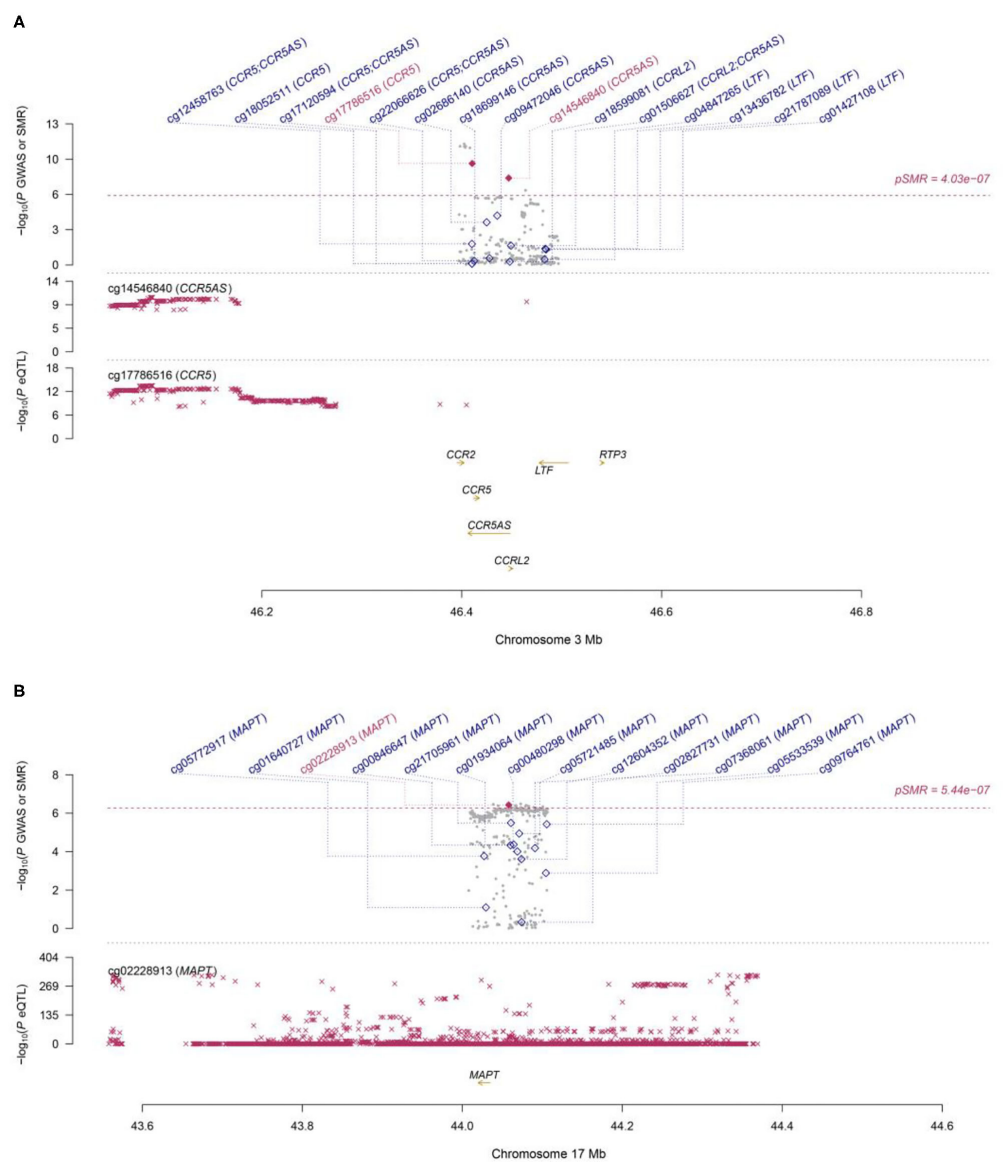
Two loci from the SMR analysis. **(A)**: Top plot, gray dots represent the *P*-values for SNPs from the COVID-19 GWAS. Bottom plots, the mQTL *P*-values of SNPs from the Hannon study for the cg14546840 and cg17786516 probes tagging CCR5 and CCR5AS, respectively. Highlighted in red are the genes (CCR5 and CCR5AS) that passed the SMR and HEIDI tests. **(B)**: Top plot, gray dots represent the *P*-values for SNPs from the COVID-19 GWAS. The bottom plot, the mQTL *P*-values of SNPs from the LBC-BSGS study for the cg02228913 probe tagging MAPT. Highlighted in red is the gene (MAPT) that passed the SMR and HEIDI tests.

Functionally important genes for COVID-19 were prioritized by the SMR analysis using six eQTL and two mQTL datasets, which identified a total of 25 associations, involving seven protein-coding genes (*TYK2, IFNAR2, OAS1, OAS3, XCR1, CCR5*, and *MAPT*) and four non-coding genes (*LRRC37A4P, IL10RB-AS1, MGC57346*, and *CCR5AS*) (Table 2; Figure 1). Several genes were implicated by two or more datasets, including *IFNAR2* (eQTL of CAGE blood, eQTL of Geuvadis lymphoblastoid cell, and mQTL of LBC-BSGS blood), *OAS1* (eQTL of Geuvadis lymphoblastoid cell and mQTL of Hannon blood), and *XCR1* (mQTL of Hannon blood and mQTL of mQTL of Hannon blood). Two protein-coding genes, *CCR5* and *MAPT*, are novel susceptibility genes for COVID-19, which were implicated by mQTL of Hannon blood and mQTL of LBC-BSGS blood, respectively (Figure 2).

**Table 2:** The SMR analyses of COVID-19.

**Type**	**Data**	**Tissue**	**Ch**	**Gene**	**Top SNP**	**P_GWAS_**	**P_eQTL_**	**Beta**	**P_SMR_**	**P_Bonferroni_**	**P_HEIDI_**	**N_SNP_**
eQTL	CAGE	blood	12	OAS3	rs7955267	1.08E-07	8.55E-33	−0.288	1.22E-06	0.010	0.089	20
eQTL	CAGE	blood	17	LRRC37A4P	rs113661667	1.01E-06	5.03E-228	−0.095	1.34E-06	0.011	0.189	20
eQTL	CAGE	blood	17	MGC57346	rs79600142	1.38E-06	4.29E-157	0.110	2.03E-06	0.017	0.375	20
eQTL	CAGE	blood	19	TYK2	rs11085727	1.27E-07	5.69E-20	−0.377	4.75E-06	0.040	0.058	15
eQTL	CAGE	blood	21	IFNAR2	rs2252639	1.08E-16	5.21E-34	0.464	7.26E-12	6.12E-08	0.017	9
eQTL	Geuvadis	Lymphoblastoid Cell	12	OAS1	rs1981555	3.10E-08	1.39E-14	−0.189	6.99E-06	0.013	0.297	16
eQTL	Geuvadis	Lymphoblastoid Cell	17	LRRC37A4P	rs62054835	1.66E-06	8.47E-28	−0.120	1.14E-05	0.021	0.016	20
eQTL	Geuvadis	Lymphoblastoid Cell	21	IFNAR2	rs2300371	5.11E-08	3.88E-14	−0.208	9.84E-06	0.018	0.098	12
eQTL	Geuvadis	Lymphoblastoid Cell	21	IL10RB-AS1	rs2300371	5.11E-08	4.93E-32	0.140	7.64E-07	1.42E-03	0.020	18
mQTL	Hannon	blood	3	NA	rs34438204	4.86E-33	1.84E-25	−12.75	3.71E-15	4.61E-10	0.031	20
mQTL	Hannon	blood	3	NA	rs13085367	6.59E-19	6.77E-15	−16.47	4.74E-09	5.88E-04	0.060	15
mQTL	Hannon	blood	3	XCR1	rs4443214	2.53E-20	1.87E-12	21.73	2.13E-08	2.64E-03	0.040	9
mQTL	Hannon	blood	3	XCR1	rs34438204	4.86E-33	7.29E-20	18.64	3.96E-13	4.91E-08	0.411	17
mQTL	Hannon	blood	3	NA	rs35110864	2.78E-19	1.06E-64	−7.46	2.08E-15	2.57E-10	0.020	20
mQTL	Hannon	blood	3	NA	rs13433997	3.26E-34	1.49E-13	−94.85	2.63E-10	3.26E-05	0.024	14
mQTL	Hannon	blood	3	NA	rs9877748	2.05E-31	4.33E-94	−5.12	3.51E-24	4.36E-19	0.018	20
mQTL	Hannon	blood	3	NA	rs35775079	1.10E-13	4.06E-14	13.07	1.17E-07	0.014	0.145	8
mQTL	Hannon	blood	3	NA	rs13069742	1.81E-31	1.10E-21	−22.65	1.38E-13	1.71E-08	0.229	18
mQTL	Hannon	blood	3	CCR5	rs7642320	6.66E-28	3.25E-14	−12.70	4.46E-10	5.54E-05	0.101	13
mQTL	Hannon	blood	3	CCR5AS	rs7642320	6.66E-28	1.67E-11	−18.20	9.76E-09	1.21E-03	0.073	7
mQTL	Hannon	blood	12	OAS1	rs10850097	1.01E-08	1.15E-30	5.63	2.92E-07	0.036	0.266	9
mQTL	LBC-BSGS	blood	17	MAPT	rs112572874	3.29E-07	0	−0.081	3.75E-07	0.034	0.070	20
mQTL	LBC-BSGS	blood	21	IFNAR2	rs2300370	7.27E-15	1.06E-35	−0.380	4.09E-11	3.76E-06	0.013	11
mQTL	LBC-BSGS	blood	3	XCR1	rs13069742	1.81E-31	8.71E-12	−0.842	3.81E-09	3.50E-04	0.413	12
mQTL	LBC-BSGS	blood	3	NA	rs71325091	1.55E-21	1.72E-10	−0.888	1.13E-07	0.010	0.055	19

*Chr, chromosome; N_SNP_, number of SNPs*.

### TWAS of COVID-19

To connect GWAS signals to tissue-specific gene expression values, the TWAS framework was used (14, 15). Inferences were made for known genetic variants in lung and whole blood tissues from the GTEx v8 expression dataset. We discovered two genes associated with the lung eQTL dataset (*CXCR6* and *CCR5*) and two genes associated with the blood eQTL dataset (*CCR9* and *PIGN*) (Table 3). Among these genes, *CCR5* and *PIGN* were novel susceptibility genes for COVID-19.

**Table 3:** Transcriptome-wide association study of the COVID-19 outcomes.

**Tissue**	**Gene**	**Z**	**P**	**P_Bonferroni_**
Lung	*CXCR6*	9.81	1.07E-22	1.46E-18
Lung	*CCR5*	6.89	5.56E-12	7.56E-08
Blood	*CCR9*	−11.33	9.09E-30	1.04E-25
Blood	*PIGN*	4.78	1.78E-06	0.02

Together, our SMR analysis and TWAS of COVID-19 identified a total of 14 genes associated with COVID-19, comprising seven genes implicated by the input or previous GWASs and seven novel genes (including three protein-coding genes, *CCR5, MAPT*, and *PIGN*).

## Discussion

Exploration of the host genetic factors contributing COVID-19 has being started as early as the first datasets became available, including ones collected in HGI. Here we present the result of our exploration of the differential susceptibility to COVID-19 in the latest HGI dataset, which we dissected using both SMR and transcriptome-wide analyses.

Genomic loci, as well as risk genes associated with the disease, were described. Notably, chromosome 3p21.31 with its chemokine receptor genes was highlighted as the peak for associations, along with chromosome 12q24.13 with the oligoadenylate synthase protein family gene cluster *OAS1, OAS2*, and *OAS3*. These enzymes activate RNAse L and degrade viral nucleic acids. The *IFNAR2* gene (21q22.11 locus) encodes a subunit for interferons alpha and beta binding receptors. Notably, *IFNAR2* is capable of producing soluble receptors, which binds and regulates endogenous production of type I IFNs (17, 18). This soluble IFNAR2 possesses both anti-proliferative and antiviral functions as well as therapeutic properties (19). In particular, in COVID-19, a protective role of *IFNAR2* has been suggested (6, 20). Another region previously associated with COVID-19 was the blood group *ABO* locus at 9q34.2 [5].

Out of 18 protein-coding genes associated with COVID-19, twelve have been reported previously (5, 6), including *ABO, CCR9, CXCR6, DPP9, FYCO1, IFNAR2, LZTFL1, OAS1, OAS2, OAS3, SLC6A20*, and *XCR1*. The present analysis uncovered six novel risk genes contributing to severe COVID-19, including *LIMD1, CCR3, CCR1, CCR2, LRRC2*, and *VSTM2A*. A majority of these six genes are located in the 3p21.31 locus (*CCR1, CCR2, CCR3, CCRL2*, and *LRRC2*), while another gene *VSTM2A* maps to newly identified loci in the 7q31.1.

Our SMR analysis identified seven protein-coding genes significantly associated with predicted expression levels in the lung or the blood. Among these genes, two are novel, including *CCR5* and *MAPT*. Our TWAS analysis identified four genes significantly associated with predicted expression levels in the lung or the blood. Two of these genes, *CCR9* and *CXCR6*, were also found within the set of COVID-19 associated genes, while *CCR5* and *PIGN* genes were novel. Together, our SMR and TWAS analyses identified three novel protein-coding genes for COVID-19, namely, *CCR5, MAPT*, and *PIGN*.

*CCR5* is located in 3p21.31 and encodes chemokine receptors expressed in macrophages and T cells. In macrophages, CCR5 protein serves as a gateway for many viruses including HIV (21). Notably, some people lack functional CCR5 allele due to 31-bp deletion within its open reading frame, and resultant loss-of-the function. In both homo- and heterozygous individuals this deletion known as rs333 is a major determinant of the resistance to HIV. It is of interest that anti-CCR5 antibody leronlimab has been tried as a post-COVID-19 therapeutic molecule and shown to downregulate both inflammatory cytokine profile and the copy number of SARS-CoV2 RNA (22). In a recent study, the CCR5-Δ32 variant was found to be significantly less frequent in hospitalized COVID-19 than in healthy controls (*P* = 0.01, OR = 0.66, 95% CI = 0.49–0.88), with no homozygotes found among the patients, compared to 1% of the controls (23). In addition, *CCR5* transcript was expressed among the patients at significantly higher levels than in the healthy non-deletion carriers (*P* = 0.01). Independent identification of *CCR5* as overexpressed in hospitalized COVID-19 patients supports the validity of our TWAS analysis.

The *MAPT* gene encodes the microtubule-associated protein tau. Genetic variation within *MAPT* was reported to be associated with multiple neurodegenerative disorders, including Parkinson's disease and Alzheimer's disease (24–26). It was also a genome-wide risk gene for autoimmune diseases and some cardiometabolic traits, including body mass index, blood cell count, osteoarthritis (25, 27–29). In addition, the gene has been implicated in interstitial lung disease (30) and lung function (31). Recently, it was shown that the tau protein binds to SARS-CoV-2 S1 receptor-binding domain (RBD) with the implication that the heparin-binding site on the S1 protein participates in the aggregation of amyloid-like proteins and promotes neurodegeneration (32). In another study, amounts of tau protein in the neuronal-enriched extracellular vesicles of patients recovering from COVID-19 were larger than in historic controls (33). In 3D human brain organoids, SARS-CoV-2 preferably targets the neurons, where it changes the distribution of Tau from axons to soma, its hyperphosphorylation, the neurotoxic death (34). Taken together, these observations point that functional variation within the tau locus may indeed be relevant to COVID-19 and especially to its neurological sequelae.

Mutations in gene *PIGN* lead to well-characterized defects in the biosynthesis of glycosylphosphatidylinositol (GPI), an anchor that tether proteins to the extracellular face of eukaryotic plasma membranes (35). Notably, genome-scale CRISPR knockout screen of cells seeded with SARS-CoV-2 and three seasonal coronaviruses (HCoV-OC43, HCoV-NL63, and HCoV-229E) highlighted glycosylphosphatidylinositol biosynthesis as one of the key dependencies for these infectious agents in two independent studies (36, 37). In the mammalian plasma membranes, GPI-anchored proteins interact with glycosphingolipids, forming dynamic microdomains. When the transfer of synthesized GPI to proteins is defective, for the lack of a particular glycosphingolipid, a lactosylceramide, the GPI biosynthesis in the endoplasmic reticulum (ER) is severely suppressed (38). The metabolism of ceramides, and lactosylceramide, in particular, is severely disturbed in SARS-Cov-2 infection, with glucosylceramide synthase inhibitors displaying marked anti-SARS-CoV-2 effects (39). It seems that GPI-glucosylceramide equilibrium may be profoundly altered by SARS-CoV-2 replication and that these changes may contribute to COVID sequelae.

The strengths of this study include the use of the largest COVID-19 dataset available. Furthermore, we have diminished the potential population heterogeneity by limiting our analysis to individuals of European ancestry. Among the noticeable study limitations are that TWAS associations are considered to be noisy and that we have limited ourselves to testing only the genetic factors associated with COVID-19 risk, rather than taking into account the social and the environmental variables as well. As the findings from our study may be relevant to the European population only, uncovered associations certainly require further validation and detailed investigation.

## Conclusions

In summary, our study highlighted some known and revealed some novel genes contributing to differential outcomes of COVID-19 disease.

## Data Availability Statement

Publicly available datasets were analyzed in this study. This data can be found here: https://www.covid19hg.org/results/.

## Author Contributions

FZ designed the study and performed the statistical analyses. FZ and AB contributed to data interpretation and wrote the manuscript. HC contributed to the data preparation. All the authors approved the final manuscript for submission and publication and agreed to be accountable for all aspects of the work.

## Conflict of Interest

The authors declare that the research was conducted in the absence of any commercial or financial relationships that could be construed as a potential conflict of interest.

## Publisher's Note

All claims expressed in this article are solely those of the authors and do not necessarily represent those of their affiliated organizations, or those of the publisher, the editors and the reviewers. Any product that may be evaluated in this article, or claim that may be made by its manufacturer, is not guaranteed or endorsed by the publisher.
