# Supplementary material for: Unraveling Risk Genes of COVID-19 by Multi-Omics Integrative Analyses
Source: Front Med (Lausanne). 2021 Sep 7;8:738687. doi: 10.3389/fmed.2021.738687 (PMC8452849; doi:10.3389/fmed.2021.738687)
Supplement: Supplementary file 1 [file Data_Sheet_1.doc]

**Supplementary File**

1. Methods

1.1 Dataset description and subject details

1.2 Genomic loci identification of the GWAS result

1.3 Summary data-based Mendelian randomization analysis

1.4 Transcriptome-wide association analysis

2. Supplementary tables

2.1 Supplementary Table 1. Genomic loci of the COVID-19 GWAS

3. Supplementary figures

3.1 Supplementary Figure 1. Manhattan plot the COVID-19 GWAS result

3.1 Supplementary Figure 2. The 3p21.31 genomic locus of the COVID-19 GWAS

4. References

**1. Methods**

**1.1 Dataset description and subject details**

Release Date: January 7, 2021.

Phenotype: Hospitalized covid vs. population, leave out 23andMe.

Samples: 9373 cases and 1197256 controls.

| **Name** | **n_cases** | **n_controls** |
| --- | --- | --- |
| BQC19_EUR | 244 | 396 |
| BelCovid_EUR | 363 | 1477 |
| CU_EUR | 453 | 2149 |
| EstBB_EUR | 90 | 196339 |
| FinnGen_FIN | 106 | 238605 |
| GENCOVID_EUR | 893 | 2443 |
| GHS_Freeze_145_EUR | 180 | 112862 |
| LGDB_EUR | 57 | 1531 |
| UCLA_EUR | 80 | 17514 |
| UKBB_EUR | 1670 | 328577 |
| idipaz24genetics_EUR | 106 | 75 |
| Amsterdam_UMC_COVID_study_group_EUR | 108 | 1413 |
| SPGRX_EUR | 311 | 302 |
| DECODE_EUR | 89 | 274322 |
| MVP_EUR | 436 | 2180 |
| HOSTAGE_EUR | 1610 | 2205 |
| BoSCO_EUR | 212 | 512 |
| FHoGID_EUR | 362 | 259 |
| Ancestry_EUR | 250 | 1967 |
| SweCovid_EUR | 77 | 3748 |
| genomicc_EUR | 1676 | 8380 |

**1.2 Genomic loci identification of the GWAS result**

FUMA was used to map SNPs to genes and identify LD-independent genomic regions.(1) Firstly, independent signiﬁcant SNPs (IndSigSNPs) were identiﬁed on the basis of their *P*-value being genome-wide signiﬁcant (*P* ≤ 5.0 × 10^−8^) and being independent from each other (r^2^ < 0.6). Secondly, Lead SNPs were identiﬁed as a subset of the independent signiﬁcant SNPs that were in LD with each other at r^2^ < 0.1 within a 1 Mb window. Genomic risk loci were identified by merging lead SNPs if they were closer than 500 kb apart. Clumping procedures were carried out on the basis of the European 1000 Genomes Project phase 3 reference panel. Due to extensive LD, the MHC region was merged into one region (chr6:25-35Mb). Genes within 100 kb of each variant were mapped.

**1.3 Summary data-based Mendelian randomization analysis**

The SMR method evaluates whether GWAS association signals are mediated by gene expression. Four eQTL and two mQTL datasets were used for the SMR analysis. The eQTL and mQTL datasets were downloaded at https://cnsgenomics.com/software/smr/#DataResource. In the SMR analysis, cis-eQTL or mQTL genetic variants were used as the instrumental variables (IVs) for gene expression.

**1.4 Transcriptome-wide association analysis**

Transcriptome-wide association study (TWAS) was conducted for the GWAS results. S-PrediXcan (2, 3) was used to calculate the gene-level association results from GWAS summary statistics and prediction models.

**2. Supplementary tables**

**2.1 Supplementary Table 1. Genomic loci of the COVID-19 GWAS**

| **Loci** | **IndSigSNPs** | **Genes** |
| --- | --- | --- |
| 1 | rs35081325;rs75826707;rs2191031;rs35605052;rs71325101;rs4411920;rs115102354;rs1542756;3:45889921:A:T;rs187726344;rs17213127 | LIMD1;SLC6A20;LZTFL1;CCR9;FYCO1;CXCR6;XCR1;CCR3;FLT1P1;CCR1;UQCRC2P1;CCR2;LRRC2 |
| 2 | rs622568 | VSTM2A |
| 3 | rs920065566 | ABO |
| 4 | rs2660 | OAS1;OAS3;OAS2 |
| 5 | rs2109069 | DPP9 |
| 6 | rs13050728;rs17860220;rs1131964 | IFNAR2 |

**3. Supplementary figures**

**3.1 Supplementary Figure 1. Manhattan plot the COVID-19 GWAS result.** The x-axis is chromosomal position of SNPs and the y-axis is the significance of the SNPs (-log10P).


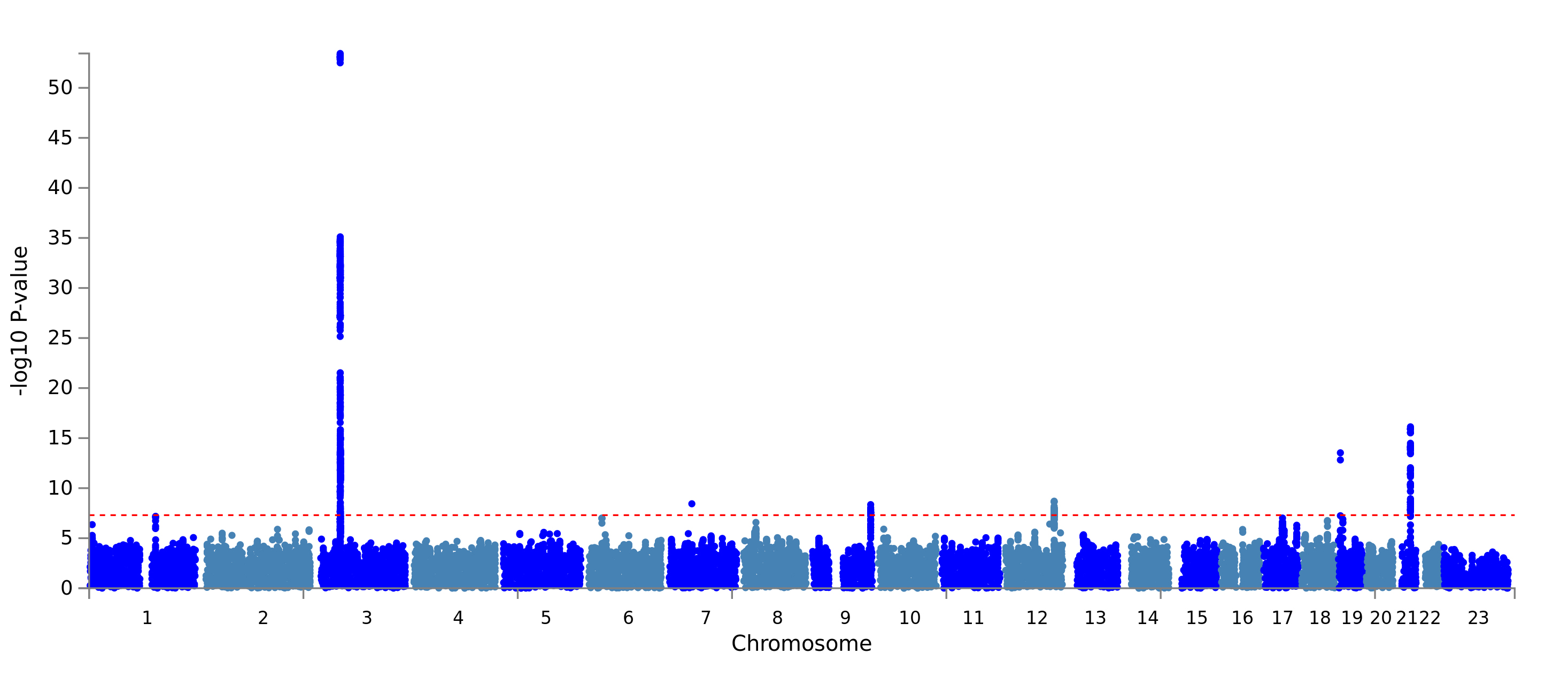


**3.1 Supplementary Figure 2. The 3p21.31 genomic locus of the COVID-19 GWAS.** Each SNP is color-coded based on the highest r^2^ to one of the independent significant SNPs, if that is greater or equal to the r^2^ threshold of 0.1. Other SNPs (below the r^2^ of 0.1) are colored in grey. The top SNPs in genomic risk loci and independent significant SNPs are circled in black and colored in dark-purple and purple, respectively. Red lines: Genes mapped by positional mapping (mapped genes). Blue lines: Non-mapped protein-coding genes. Dark grey lines: Non-mapped non-coding genes.


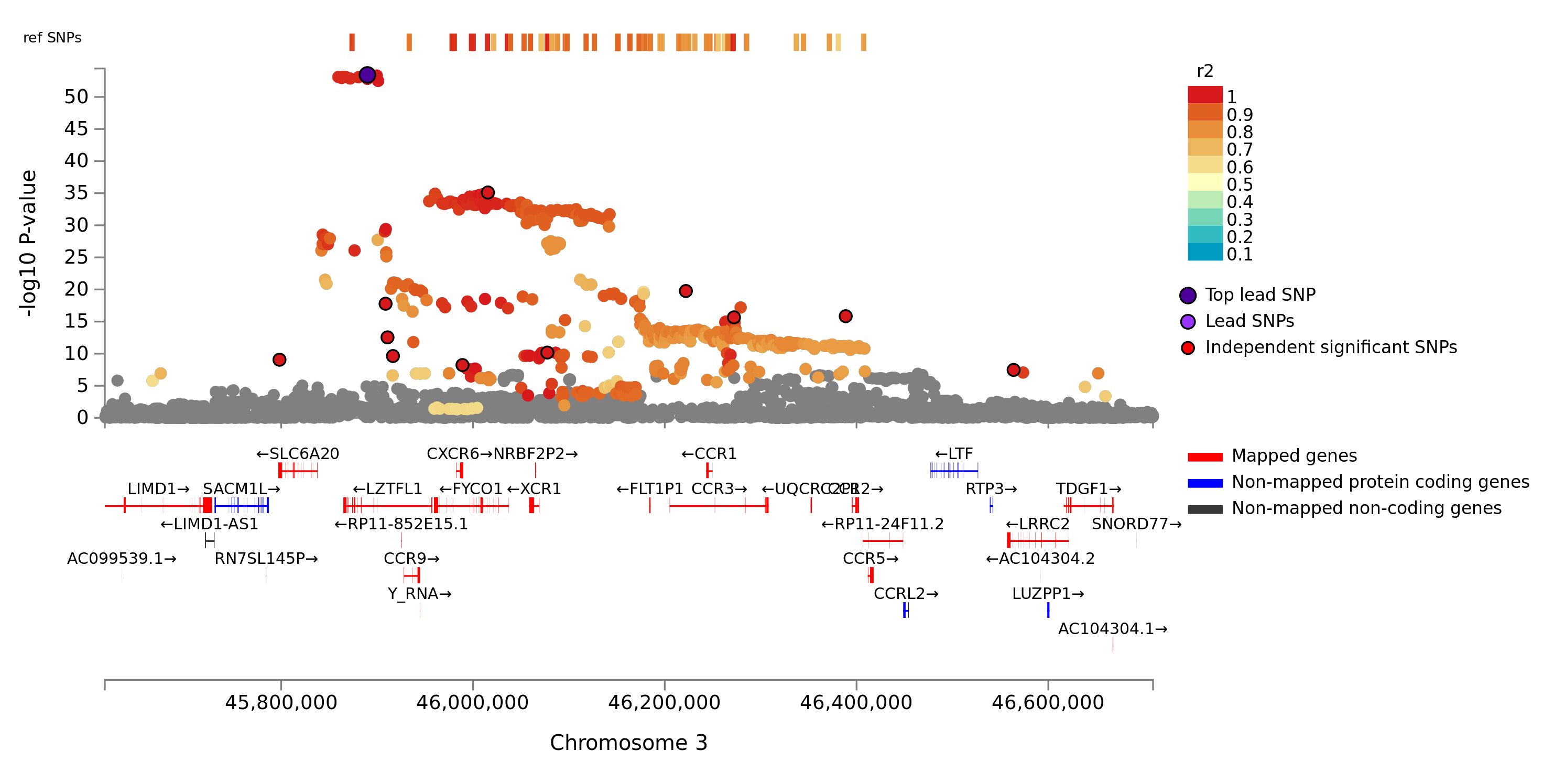


**4. References**

1. Watanabe K, Taskesen E, van Bochoven A, Posthuma D. Functional mapping and annotation of genetic associations with FUMA. *Nat Commun* (2017) 8(1):1826. doi: 10.1038/s41467-017-01261-5

2. Gamazon ER, Wheeler HE, Shah KP, Mozaffari SV, Aquino-Michaels K, Carroll RJ, et al. A gene-based association method for mapping traits using reference transcriptome data. *Nat Genet* (2015) 47(9):1091-1098. doi: 10.1038/ng.3367

3. Barbeira AN, Dickinson SP, Bonazzola R, Zheng J, Wheeler HE, Torres JM, et al. Exploring the phenotypic consequences of tissue specific gene expression variation inferred from GWAS summary statistics. *Nat Commun* (2018) 9(1):1825. doi: 10.1038/s41467-018-03621-1
